# Micro-incision vitrectomy surgery for primary rhegmatogenous retinal detachments with posterior vitreous detachments in elderly patients: Preoperative characteristics and surgical outcomes

**DOI:** 10.1371/journal.pone.0244614

**Published:** 2021-01-06

**Authors:** Kazuya Yamashita, Saki Sakakura, Yoshiko Ofuji, Maho Sato, Takashi Nagamoto, Hirohisa Kubono, Mari Kawamura, Kotaro Suzuki

**Affiliations:** Department of Ophthalmology, Keiyu Hospital, Yokohama, Japan; University of Toronto, CANADA

## Abstract

**Purpose:**

To assess the preoperative characteristics and surgical outcomes of using micro-incision vitrectomy surgery (MIVS) to treat RRD with posterior vitreous detachment (PVD) in an older and a younger patient group.

**Methods:**

This retrospective cohort study included 407 eyes from 397 patients with primary RRD with PVD who were consecutively treated in our hospital from February 2016 to February 2020. PVD was diagnosed clinically by the presence of a Weiss ring, or was diagnosed morphologically via optical coherence tomography and subsequently confirmed during surgery. The main outcome measures were preoperative RRD characteristics, best-corrected visual acuity (BCVA), and postoperative complications.

**Results:**

Data were analysed from 55 eyes in the elderly group (age 70 and older), and 352 eyes in the young group (age 69 and younger). There was no significant inter-group difference in the initial reattachment rate. Preoperative characteristics indicated that elderly patients had a significantly lower rate of phakic eyes, shorter mean axial length, lower lattice incidence, and longer time spans from onset to surgery. There were no significant between-group differences in the incidence of the following complications: fibrin formation, intraocular pressure elevation, epi-retinal membrane on the macula, intraocular lens optic capture, proliferative vitreoretinopathy, and vitreous haemorrhage. While the elderly patients had significant postoperative improvements in BCVA, these improvements were significantly lower than those of the younger patients.

**Conclusions:**

This study highlighted the characteristics and surgical outcomes of MIVS in elderly patients with RRD. Although the time from onset to surgery was longer, MIVS still can be performed safely to improve older patients’ postoperative BCVA.

## Introduction

Rhegmatogenous retinal detachment (RRD) can lead to blindness without appropriate and timely treatment. Posterior vitreous detachment (PVD) caused by senile vitreous liquefaction is the main mechanism for RRD in elderly patients [1]. However, most developing countries with limited medical resources do not prioritise RRD treatment [2]. This may primarily be attributed to the occasional need for multiple surgeries and the fact patients need to adhere to specific postoperative positional restrictions [3]. The annual incidence of RRD is low, ranging from 6.3 to 17.9 per 100,000 individuals in the global population [4]. The estimated costs of pars plana vitrectomy (PPV) surgery to treat RRD are high ($7,940 USD in America) [5]. The RRD post-surgery vision-related quality of life also is significantly impaired in these patients [6]. Partial-thickness retinal folds related to postoperative metamorphopsia are common after PPV for RRD repair [7]. Most significantly, patients of advanced age often have inferior functional results (i.e., best-corrected visual acuity [BCVA]), compared to that of younger patients after this operation [8]. Therefore, in clinical practice, elderly people often decline the surgery in view of their relatively few remaining years of life. However, a recent study reported that RRD surgery not only improved the visual acuity and quality of life of elderly patients aged >70 years but also was cost effective despite the relatively shorter life expectancies of the participants [9].

Longer life expectancies likely contribute to rising myopia rates, the growing popularity of cataract surgery and the increasing number of elderly patients suffering from RRD [4]. Scleral buckling (SB) and PPV are common RRD treatments. Although a previous study evaluated SB versus PPV for RRD [10], a recent comprehensive study found that patients age 65 years and older had the greatest overall PPV treatment rates [11]. Moreover, Anteby et al. reported that vitrectomy is effective for improving visual function even in individuals aged ≥85 years [12]. The PPV has evolved to micro-incision vitrectomy surgery (MIVS), which involves smaller, sutureless sclerotomy wounds that result in a comfortable procedure without serious postoperative complications [13].

The main mechanism for RRD in elderly people is thought to be the vitreous remaining attached to the retina during PVD progression, resulting in a horseshoe retina tear [14]. Therefore, ophthalmic surgeons should select appropriate RRD treatments for their elderly patients only after considering this RRD mechanism. Data from the UK’s National Ophthalmology Database have been evaluated to determine the results of PPV for RRD in patients with a mean age above 60 years [15]. However, the differences in surgical outcomes of using MIVS to treat RRD with PVD between young patients and elderly patients aged >70 years remain unclear. When studying this topic with elderly people, it is difficult to establish a control group. Furthermore, patients recommended for PPV who were initially too frail for surgery tend not to be included in such studies. For these reasons, it is worthwhile to document the preoperative characteristics and surgical outcomes of young patients and elderly patients aged >70 years with RRD during PVD progression. This study used a consecutive retrospective analysis to compare the characteristics of older and younger patients with RRD and PVD as well as their outcomes after MIVS.

## Patients and methods

### Patients

We analysed retrospectively the medical records of 407 eyes of 397 consecutive patients who underwent MIVS to treat RRD with PVD. All patients were followed for more than 3 months. We assessed the PVD preoperatively by Weiss ring or swept-source optical coherence tomography (SS-OCT; DRI OCT-1 Triton; Topcon, Tokyo, Japan) and confirmed it during surgery. This study was approved by the Ethics Committee of Keiyu Hospital, Kanagawa, Japan (Approval Number: R2-49) and conducted from February 2016 to February 2020. All data were fully anonymised before we accessed them and the IRB waived the requirement for informed consent. Three surgeons (HK, KM and KS) performed all surgical procedures. The patients in this case series were divided into two groups: the young patient group (YG; under age 69) and the elderly patient group (EG; over age 70). This study was performed in accordance with the Declaration of Helsinki.

### Exclusion criteria

We excluded the diagnosis of RRD in elderly patients with a history of severe systemic diseases, such as the tractional retinal detachment by diabetic retinopathy or retina vascular occlusive diseases. Of the 458 consecutive RRD eyes screened, 51 eyes were excluded. These patients were excluded for the following reasons (the numbers in parentheses indicate the number of eyes excluded): the history of vitrectomy or placement of a scleral buckle (13), incomplete PVD (30), proliferative vitreoretinopathy of grade C or worse (7) or myopic macular hole (1). After these exclusions, 407 eyes were included in this retrospective study. S1 File shows demographic data and outcomes of vitrectomy in this series. S1 File contains data from 407 cases of RRD included in this study.

### MIVS

MIVS procedures were performed using a 25- or 27-gauge system (Constellation, Alcon Laboratories Inc., Fort Worth, TX, USA) with a three-port PPV employing a wide-angle viewing system (Resight, Carl Zeiss Meditec, Oberkochen, Germany). We also completed simultaneous cataract surgery in which we implanted phacoemulsification and intraocular lens [IOL] in cataract-affected eyes. We ascertained the presence of PVD by injecting triamcinolone acetonide (MaQaid, Wakamoto Pharmaceutical, Tokyo, Japan) in the posterior retinal surface after core vitrectomy. We shaved the peripheral vitreous as much as possible with scleral indentation. We used liquid perfluorocarbon (Perfluoron, Alcon Laboratories Inc.) to stabilise the detached retina in some eyes. We also performed internal limiting membrane (ILM) peeling with staining by brilliant blue-green (BBG; Sigma-Aldrich Co., St Louis, MO, USA) in some cases. We completed fluid-air exchange and photocoagulation around all retinal tears and lattice after vitreous shaving. Finally, we executed an air, gas (20% sulfur hexafluoride [SF6], 14% octafluoropropane [C3F8]) or silicon oil exchange after the retina was reattached completely. Characteristics of the surgical procedure in the young and elderly patients are presented in Table 3.

### After MIVS

All patients were instructed to remain in a prone position after vitrectomy until the next morning. Some patients were instructed to avoid maintaining the original retinal breaks in the lowest position. For example, patients with an inferior break were instructed to maintain a supine position, while patients with a lateral break were instructed to maintain a lateral position. After vitrectomy day one, most patients were instructed not to maintain a specific posture during the daytime and to avoid a supine position to prevent optic IOL capture during asleep. Some patients continued to have some movement restrictions until a daily examination showed sufficient retinal photocoagulation scarring.

### Statistical analysis

Continuous values were expressed as the mean ± standard deviation. BCVA was converted to the logarithm of the minimal angle of resolution (log MAR) values for all calculations. Data are presented as mean ± SD and were compared using the Mann–Whitney U test, Chi-squared test and Friedman test using the *R* statistical programming language (R version 4.0.2; The Foundation for Statistical Computing, Vienna, Austria). A p value of <0.05 was considered statistically significant.

## Results

### Age distribution of RRD with PVD patients

**Fig 1:**
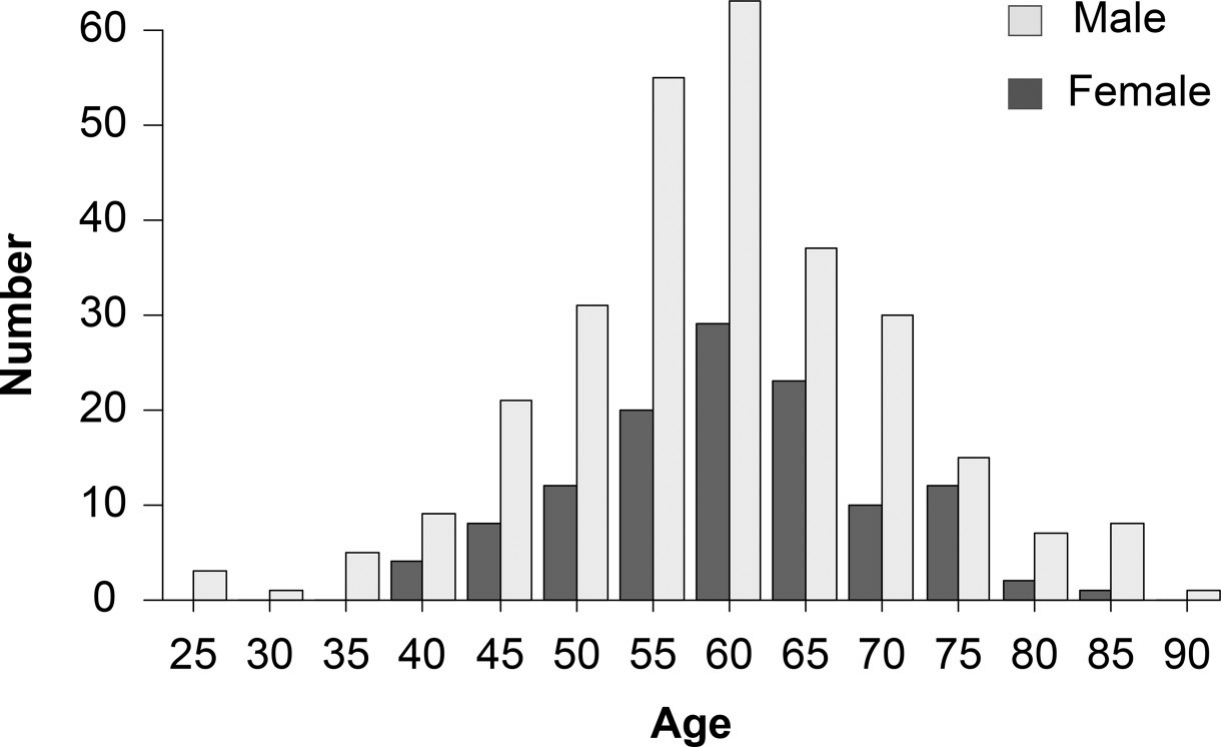
Distribution of Rhegmatogenous Retinal Detachments (RRD) with Posterior Vitreous Detachment (PVD) in different age groups.

The 407 eyes of 397 patients (age 57.6 ± 10.6) with RRD were followed for a mean of 9.3 ± 10.1 months. Table 1 and Fig 1 show the distribution of RRD with PVD in different age groups. The age distribution showed a single peak pattern with a peak at 55–59 years (63 men and 29 women). In both age groups, the majority of patients were men.

**Table 1:** Distribution of RRD with PVD in different age groups.

	20–25	25–30	30–35	35–40	40–45	45–50	50–55	55–60	60–65	65–70	70–75	75–80	80–85	85–90
Female (No.)	0	0	0	4	8	12	20	29	23	10	12	2	1	0
Male (No.)	3	1	5	9	21	31	55	63	37	30	15	7	8	1

The age distribution showed a single peak pattern with a peak at 55–59 years (63 men and 29 women). In both age groups, the majority of patients were men.

### BCVA changes in patients with RRD with PVD

**Fig 2:**
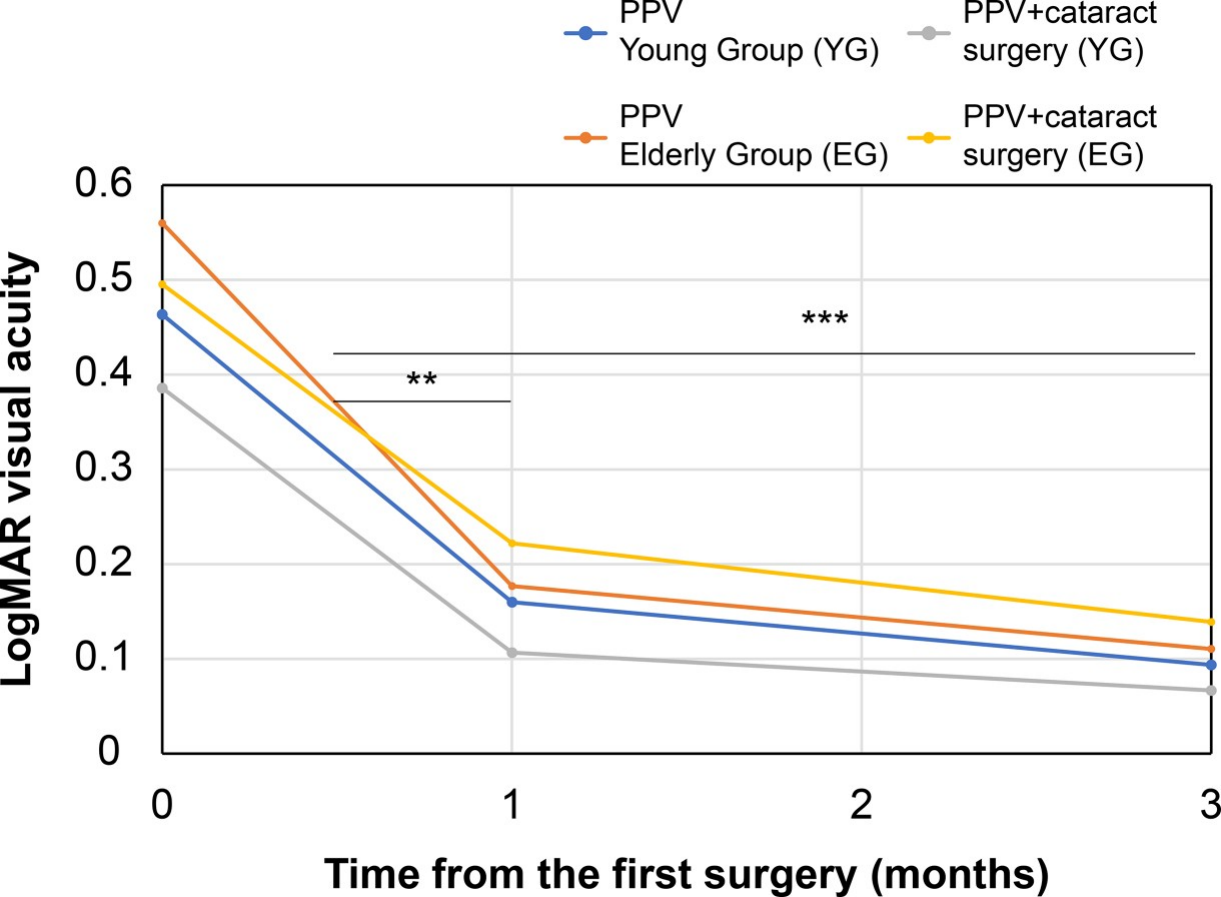
Changes in Best-Corrected Visual Acuity (BCVA) in patients with RRD with PVD.

Fig 2 shows a comparison of the BCVA preoperatively, after one month and after three months. Comparison of the preoperative BCVA values revealed that all patients treated with PPV did not show inter-group significant differences (0.56 ± 0.65 in the young group and 0.46 ± 0.68 in the elderly group, p = 0.349). Similarly, patients with PPV with cataract surgery did not show significant differences (0.50 ± 0.65 in the young group and 0.39 ± 0.63 in the elderly group, p = 0.191). All patients in both groups (treated by PPV or PPV with cataract surgery) had significant postoperative improvements one month after surgery (PPV in YG: p = 3.5 × 10^−5^; PPV in EG: p = 0.0099; PPV with cataract surgery in YG: p = 9.3 × 10^−16^; PPV with cataract surgery in EG: p = 0.0066). Similarly, All patients in both groups (treated by PPV or PPV with cataract surgery) had significant postoperative improvements three months after surgery (PPV in YG: p = 1.2 × 10^−7^; PPV in EG: p = 0.0026; PPV with cataract surgery in YG: p = 2.0 × 10^−16^; PPV with cataract surgery in EG: p = 0.0012). Comparing the BCVA after one month and after three months, elderly group patients treated with PPV and PPV with cataract surgery did not show significant improvements (PPV in EG: p = 0.0633; PPV with cataract surgery in EG: p = 0.1065). In contrast, young group patients treated with PPV and PPV with cataract surgery showed significant improvements (PPV in YG: p = 3.9 × 10^−6^; PPV with cataract surgery in YG: p = 5.7 × 10^−7^). While the BCVA for both groups improved postoperatively, the elderly group’s BCVA did not improve significantly from one month to three months after surgery.

Comparing the log MAR visual acuity pre-operation and after one month or three months. Comparing the log MAR visual acuity preoperatively and one month after vitrectomy, all cases of young and the elderly group treated by micro-incision and pars plana vitrectomy (PPV) with cataract surgery had significant improvement postoperatively (*p* < 0.01). Comparing the log MAR visual acuity preoperatively and after vitrectomy three months, all cases (young and elderly groups treated either by PPV or PPV with cataract surgery) had significant postoperative improvement (*p* < 0.005). Comparing the log MAR visual acuity after one month and after three months, the elderly group (treated with either PPV or PPV with cataract surgery) showed no significant improvement (PPV in EG: p = 0.0633; PPV with cataract surgery in EG: p = 0.1065). However, the young group (treated with both PPV and PPV with cataract surgery) showed significant improvement (PPV in YG: p = 3.9 × 10^−6^; PPV with cataract surgery in YG: p = 5.7 × 10^−7^; ** *p* < 0.01, *** *p* < 0.005).

Abbreviation: log MAR, the logarithm of the minimum angle of resolution.

### Baseline patient characteristics

Table 2 shows preoperative characteristics for both groups. There were 286 (70.3%) eyes belonging to the male participants and 121 (29.8%) eyes to the female participants. Furthermore, 212 (52.1%) of the eyes were right eyes. The mean day from onset to surgery was 7.7 ± 6.5 for the total sample. However, this timespan was significantly longer in the elderly group (10.3 ± 7.3 days) compared to the young group (7.4 ± 6.4 days). The mean axial length was 26.1 ± 1.8 mm for the entire sample. The elderly group’s mean axial length (24.6 ± 1.4 mm) was significantly shorter than that of the young group (26.3 ± 1.8 mm). In 164 eyes (40.3%), the macula was detached preoperatively. Additionally, 68 eyes were pseudo-phakic and 339 eyes were phakic. The phakic eye rate of the individuals in the elderly group (33 eyes, 60.0%) was significantly lower than that of the young group (310 eyes, 88.1%). For the total sample, the mean number of quadrants affected was 1.81 ± 0.77 and the mean number of breaks was 2.04 ± 1.37. The most frequent location for the largest break was superior-temporal (224 eyes, 55.0%). The least frequent location of the largest break was inferior-nasal (28 eyes, 6.9%). Lattice was found in 205 eyes (50.4%) in the total sample. Lattice was found in 20 eyes (36.4%) in the elderly group, a number significantly less than that of the young group (185 eyes, 52.6%). For the total sample, the mean preoperative intraocular pressure (IOP) was 12.5 ± 3.2 mmHg and the mean preoperative BCVA was 0.42 ± 0.65 (logMAR). There was no significant inter-group difference in the preoperative BCVA. Preoperative characteristics indicated that as compared to younger patients, the elderly patients had a significantly lower rate of phakic eyes, shorter mean axial length, lower lattice incidence and longer time spans from onset to surgery.

**Table 2:** Baseline patient characteristics in elderly and younger groups.

	young (n = 352)	elderly (n = 55)	P value
Male [No. (%)]	248 (70.5)	38 (69.1)	0.962
Right Eye [No. (%)]	182 (51.7)	30 (54.5)	0.805
Day from onset to surgery [days (mean ± SD)]	7.4 ± 6.4	10.3 ± 7.3	0.0025
Axial length [mm (mean ± SD)]	26.3 ± 1.8	24.6 ± 1.4	4.6 × 10^−11^
Macular status on [No. (%)]	215 (61.1)	28 (50.9)	0.20
Lens status Phakic [No. (%)]	310 (88.1)	33 (60.0)	3.1 × 10^−7^
Extent of detached retina (quadrants)			
1	136 (38.6)	18 (32.7)	
2	168 (47.7)	23 (41.8)	
3	36 (10.2)	12 (21.8)	
4	12 (3.4)	2 (3.6)	
(mean ± SD)	1.78 ± 0.76	1.96 ± 0.83	0.119
Number of breaks			
1	160 (45.5)	31 (56.4)	
2	96 (27.3)	15 (27.3)	
3+	96 (27.3)	9 (16.4)	
(mean ± SD)	2.10 ± 1.4	1.65 ± 0.88	0.0546
Location of the largest break			
Superior-Temporal	190 (54.0)	34 (61.8)	0.347
Superior-Nasal	85 (24.1)	9 (16.4)	0.271
Inferior-Temporal	54 (15.3)	7 (12.7)	0.763
Inferior-Nasal	23 (6.5)	5 (9.1)	0.682
Presence of lattice [No. (%)]	185 (52.6)	20 (36.4)	0.0367
Preoperative IOP [mmHg (mean ± SD)]	12.6 ± 3.2	11.5 ± 2.9	0.0861
Preoperative BCVA [logMAR (mean ± SD)]	0.41 ± 0.64	0.52 ± 0.65	0.081

Abbreviation: IOP: IntraOcularPressure; BCVA: Best-Corrected Visual Acuity.

### Surgical procedure characteristics

Table 3 shows the surgical procedure characteristics. Combined cataract surgery during vitrectomy was performed in 277 eyes (68.1%), occurring significantly less frequently in the elderly group (30 eyes, 54.5%) compared to the young group (247 eyes, 70.2%). We performed the combined cataract surgery during vitrectomy in 30 of the 33 phakic eyes in the elderly group. Combined SB during vitrectomy was performed in 11 eyes (2.7%). ILM peeling during vitrectomy was performed in 83 eyes (20.4%). MIVS was performed using a 25-gauge system (240 eyes, 59.0%) or a 27-gauge system (167 eyes, 41.0%). The total operation time was 98.4 ± 45.0 minutes. An air (174 eyes, 42.8%), gas (using 20% sulfur hexafluoride [SF6; 215 eyes, 52.8%] or perfluoropropane [C3F8; 8 eyes, 2.0%]) or silicon oil (10 eyes, 2.5%) exchange was performed. Except for combined cataract surgery during vitrectomy, there was no significant “inter-group difference” in surgical procedure characteristics.

**Table 3:** Characteristics of the surgical procedure in the young and elderly patients.

	young (n = 352)	elderly (n = 55)	P value
Combined cataract surgery during vitrectomy [No. (%)]	247 (70.2)	30 (54.5)	0.0311
Combined SB during vitrectomy [No. (%)]	10 (2.8)	1 (1.8)	1
Combined ILM peeling during vitrectomy [No. (%)]	69 (19.6)	14 (25.5)	0.411
25-gage system vitrectomy [No. (%)]	212 (60.2)	28 (50.9)	0.246
27-gage system vitrectomy [No. (%)]	140 (39.8)	27 (49.1)	0.246
Total operation time [minute (mean (mean ± SD)]	98.3 ± 43.4	99.5 ± 54.0	0.567
Air exchange [No. (%)]	146 (41.5)	28 (50.9)	0.243
20% sulfur hexafluoride [SF6] exchange [No. (%)]	192 (54.5)	23 (41.8)	0.107
Perfluoropropane [C3F8] exchange [No. (%)]	5 (1.4)	3 (5.5)	0.138
Silicone oil exchange [No. (%)]	9 (2.6)	1 (1.8)	0.931

Abbreviation: SB: Scleral Buckling; ILM: Internal Limiting Membrane.

### Postoperative outcome measures

Table 4 shows postoperative outcome measures. There was no significant difference in follow-up periods between the young group (9.3 ± 10.2) and the elderly group (8.9 ± 9.7). Initial reattachment occurred in 379 (93.1%) eyes, with no significant between-group difference in the initial reattachment rate. One month after the vitrectomy, the elderly group’s BCVA (0.20 ± 0.42, log MAR) was significantly worse than that of the young group (0.12 ± 0.32, log MAR). Similarly, three months after the vitrectomy, the elderly group’s BCVA (0.16 ± 0.34, log MAR) also was significantly worse than the young group’s BVCA (0.084 ± 0.30). There were no significant differences between the two groups in the occurrence of the following general complications after vitrectomy: fibrin formation, IOP elevation, IOL optic capture, ERM on macula, vitreous haemorrhage and proliferative vitreoretinopathy. Despite the similar complications rates between the two groups, the elderly group’s postoperative BCVA was worse than the young group’s BCVA.

**Table 4:** Postoperative outcome measures in elderly and younger groups.

	young (n = 352)	elderly (n = 55)	P value
Follow-up [month (mean ± SD)]	9.3 ± 10.2	8.9 ± 9.7	0.875
Fibrin formation in AC on postoperative day 1 [No. (%)]	34 (9.7)	4 (7.3)	0.752
IOP elevation (>22mmHg) [No. (%)]	67 (19.0)	7 (12.7)	0.347
BCVA 1 month after vitrectomy [logMAR (mean ± SD)]	0.12 ± 0.32	0.20 ± 0.42	0.0447
BCVA 3 month after vitrectomy [logMAR (mean ± SD)]	0.074 ± 0.29	0.13 ± 0.31	0.0211
IOL optic capture after vitrectomy [No. (%)]	16 (4.5)	2 (3.6)	1
ERM on macula after vitrectomy [No. (%)]	47 (13.4)	5 (9.1)	0.507
VH after vitrectomy [No. (%)]	7 (2.0)	1 (1.8)	1
Initial reattachment [No. (%)]	328 (93.2)	51 (92.7)	1
PVR after vitrectomy [No. (%)]	3 (0.85)	0 (0)	1
Final reattachment [No. (%)]	352 (100)	55 (100)	1

Abbreviations: AC: Anterior chamber; IOP: Intraocular pressure; ERM: Epi-retinal membrane; VH: Vitreous haemorrhage; PVR: proliferative vitreoretinopathy.

## Discussion

As previously mentioned, the main mechanism for RRD in elderly people likely is the vitreous remaining attached to the retina during PVD progression, causing a horseshoe retina tear [14]. It is well known that the vitreous becomes more liquefied with age. Ocular saccades can cause vitreous fluid movement that forces fluid into the subretinal space, thereby dissecting the neurosensory retina off of the retinal pigment epithelium, especially when the break is held open by vitreous traction [16, 17]. Considering this mechanism of RRD, releasing vitreous traction with PPV seems to be an effective treatment, even in elderly patients who are in poor general health compared to their younger counterparts. To our knowledge, this is the first study to use consecutive retrospective analysis to document and compare the characteristics and MIVS outcomes in elderly and younger populations who have RRD with PVD.

The second peak of RRD incidence in the young age group is known as a typical differential feature of the East Asian population [18], which has not been observed in the Western population [19]. However, Sakamoto et al. recently reported that the RRD population in Japan does not have a two-peak pattern, but rather a single peak, which is due to the rapid ageing of the Japanese population [20]. If the second peak also was not found in Western reports, RRD prevalence would be expected to increase with age. Considering our ageing society, clinicians should remain alert and be prepared to treat a large number of older patients with RRD.

The elderly group’s phakic eye ratio was significantly lower than that of the young group. Additionally, the proportion of RRD occurring after cataract surgery has increased in older individuals in Japan, rising from 4.7% in 1990 [21] to 18.2% in 2016 [20]. Because PVD is more likely to occur after cataract surgery, postoperative PVD should be considered an important risk factor for RRD [22]. In the elderly group, the mean axial length was significantly shorter and the presence of lattice was significantly lower than the same measures in the young group. In myopic eyes, large eyeball volume induces RRD through the earlier occurrence of PVD, lattice degeneration and thinner retinas than those observed in emmetropic eyes [4, 14]. This result supported myopia-induced early vitreous detachment as a major mechanism of RRD occurrence in young East Asian patients, while senile vitreous liquefaction and detachment remains the main RRD mechanism in elderly patients [1]. Interestingly, the mean days from onset to surgery in the elderly group was significantly longer than in the young group. Possible factors delaying diagnosis in elderly patients with RRD include vitreous gel liquefaction [23], impaired macular function [24] and the presence of cataract [25]. Furthermore, the interval from onset to surgery was significantly associated with the area of RRD [20]. Asking patients about subjective symptoms, such as visual field loss associated with RRD, is important for promoting early detection of this condition.

Although three different surgeons performed the MIVS procedures, there was no significant “inter-group difference” in surgical procedure characteristics. Interestingly, total operation time, even in the elderly group, remained almost the same as the time noted in a previous study [26]. Some elderly patients have issues that complicate matters during and after surgery (e.g., not being able to lie flat for a long time because of neck or back problems and being hard of hearing) [27]. However, the elderly patients in this study tolerated MIVS well when it was performed in the supine position with local anaesthesia. In eyes filled with gas, inflammatory cells and cytokines should accumulate inferiorly after vitrectomy, which could contribute to ERM formation and fibrin in patients’ eyes (depending on their position) [28]. However, there were no “between-group differences” in post-MIVS complications, and our current rate of ERM development was comparable to rates reported in previous studies [29]. The overall primary retinal attachment rate in this study was 93.1%, which compares favourably with previously reported rates [10, 28, 30–32]. The latest PPV advances—MIVS, 25-gauge or 27 gauge sizes and wide-angle viewing systems—help surgeons successfully perform less invasive surgeries. Even elderly people can undergo this surgery without significant problems.

Our results documented significant BCVA improvements from preoperative and to post-vitrectomy measurements (with postoperative retina attachment), even the elderly group (treated by PPV or PPV with cataract surgery; Fig 2). The improvements in macular function and BCVA may be attributed to changes in the vitreous cytokines or recovery of the retinal ischaemia [33]. Our findings highlighted comparable visual improvements in RRD cases, regardless of the type of surgery [33], as well as reduced macular function, even in eyes with macula-on retinal detachment [34]. However, our results also showed that the post-vitrectomy BCVA in the elderly patients was significantly poorer than that of the young patients. Despite the absence of a significant inter-group difference in the preoperative BCVA in the current study, the elderly patients had a longer time span from onset to surgery. Studies have previously reported that the number of human photoreceptor cells decreased considerably after RRD and continued to decrease with time [35], and the presence of macular dysfunction in eyes even macula-on RRD [34]. A relatively longer duration from diagnosis to surgery is associated with a poorer prognosis of vision. Alternatively, Baba et al. reported that the postoperative BCVA of the elderly patients treated for RRD within 1 week of the diagnosis remained poor [33]. These findings suggest an inferior clinical activity of a potentially low macular function that is associated with ageing. Based on acuity testing in younger patients, Ross et al. reported that visual function may continue to improve in the long term [36]. Our findings showed that, while the BCVA of the elderly group (including patients treated with PPV or PPV with cataract surgery) improved from one month to three months after surgery, the improvement was not significant. However, we were not able to conclude whether age is a negative predictor for postoperative visual outcomes. Ma et al. reported that older age had negative effects on preoperative BCVA and the BCVA of elderly patients (age 70 years and older) increased continuously from three months to one year after surgery [9]. In contrast, Heussen et al. observed no relationship between age and inferior visual outcomes [37]. However, the results from all of these studies cannot be directly compared due to the participants’ different baseline conditions and the fact that BCVA generally improves after surgery, even in older patients.

The major limitations of the current study were its relatively small sample size, retrospective data collection, short follow-up period and non-randomised study design. Additionally, the selection of surgery type, tamponade gas or silicon oil was made according to the surgeon’s experience.

## Conclusion

The results of this retrospective study suggest that MIVS can be used successfully to treat younger patients and as well as those age 70 and older without complications. Even elderly patients have significantly improved postoperative BCVA. These findings can serve as the basis for randomised and prospective studies with larger sample sizes designed to further investigate elderly patients characteristics’ for the purpose of managing RRDs.

## Supporting information

S1 File Demographic data and outcomes of MIVS in this series. S1 File contains data from 407 cases of RRD included in this study.
(XLSX)
